# Knowledge, attitudes and practices on tuberculosis infection prevention and associated factors among rural and urban adults in northeast Tanzania: A cross-sectional study

**DOI:** 10.1371/journal.pgph.0000104

**Published:** 2021-12-08

**Authors:** Method Kazaura, Switbert Rwechungura Kamazima

**Affiliations:** 1 Department of Epidemiology and Biostatistics, Muhimbili University of Health and Allied Sciences, Dar es Salaam, Tanzania; 2 Behavioral Sciences Department, Muhimbili University of Health and Allied Sciences, Dar es Salaam, Tanzania; University of California Irvine, UNITED STATES

## Abstract

Almost 10 million of the global population was infected with tuberculosis (TB) in 2017. Tanzania is among countries with high incidence of TB. Although control measures of TB are multi factorial, it is important to understand the individual’s knowledge, attitudes and practices (KAP) in order to control TB infection. We conducted a cross-sectional study in northeast Tanzania; recruited and interviewed 1519 adults from two districts, one rural and another urban. We scored each participant using several questions for each construct of KAP. A study participant scoring at least 60% of the possible maximum scores was considered as having a good knowledge, positive attitude or good practices. And herein, a participant having positive TB attitude would mean they acknowledge TB exist, recognizes its impact on health and would seek or advise TB-infected individuals to seek the correct remedies. We applied multiple linear regression analysis to assess independent individual-level factors related to TB on KAP scores in the rural and urban populations. Overall, less than half (44%) of the study participants had good overall knowledge about TB infection and significantly more urban than rural adult population had good overall knowledge (*p*<0.001). Almost one in ten, (11%) of all study participants had positive attitudes towards TB infection. More urban study participants, (16%) had positive attitudes than their rural counterparts, 6%). Almost nine in ten (89%) of all study participants had good practices towards TB prevention and control; significantly more adults in urban, (97%) than the rural populations (56%) (*p*<0.01). Predictors of KAP scores were individual’s education and main source of income. Adults in rural and urban northeast Tanzania have poor knowledge, attitudes and practices for TB infection and prevention. Strategies focusing on health education are important for control of TB, especially among rural communities.

## Introduction

Human tuberculosis (TB) is a chronic infectious disease mainly caused by a family of organisms known as *Mycobacterium tuberculosis*. Although TB can either be latent or active, 90% of the active TB cases arise from the latent, commonly referred to as reactivation tuberculosis [1,2]. In 2018, it was estimated that the global incidence of TB was more than 10 million people and almost one-fifth of the cases died [3]. In view of the fact that HIV-infected people are more likely to develop TB [3,4], there are more TB cases in less developed countries where the burden of HIV&AIDS is high [5,6]. Major countries with more TB cases and deaths include Indonesia, China, Philippines, Pakistan, Nigeria and South Africa [7].

Tanzania is among sub-Saharan countries with high burden of TB [8]. In 2018, the notification rate for new and relapsed TB cases in northeast Tanzania, was about 129 per 100,000 population [9]. In 2011, the World Health Organization (WHO) estimated the prevalence of TB infection in Tanzania to be 0.2% with a more recent estimate of 295 per 100,000 adult populations [10–12]. A higher burden is also seen in children, whereby in 2016 almost 14% of children under the age of 15 years from Central and Eastern Tanzania were detected as having TB infection [13]. Data from some sub-Sahara African countries suggest higher burden of TB in urban than from the rural settings [14].

There is higher regional variability of TB prevalence across the 26 administrative regions of mainland Tanzania [15,16]. However, the TB cases notification rate rose from 2.69 per 100,000 population in 2017 to 2.81 in 2019 [16]. In order to control and reduce the burden of TB at the community or individual-level, various measures have been suggested [17,18]. At an individual level, some studies suggest sex, age, education, marital status, main sources of income and exposure to mass media are associated with knowledge, attitudes and practices (KAP) towards TB prevention and control [19–25]. In addition, adults living in urban areas have been associated with higher overall better knowledge of TB as compared to their counterparts in rural areas [26,27].

Understanding these KAP factors at the community and individual levels is very important especially when planning the implementation and even evaluation of programs aimed at preventing and controlling TB infection [28]. Nevertheless, to the best of our search, there are limited current data in Tanzania that focus on KAP towards tuberculosis. The available data are old, not specific to the study population or beyond examining KAP towards prevention and control of TB infection. Therefore in this study, we assessed KAP about TB infection prevention and control among adults in rural and urban areas of northeast Tanzania and determined their associated individual-level factors that are essential for appropriate design of control measures against TB infection in Tanzania.

## Materials and methods

### Study design and settings

We conducted a cross-sectional analytical study design in Tanga region, northeast Tanzania. We purposefully selected Tanga because it is one of the regions with higher TB case notification rates than the national average of 1.38 per 100,000 population [16]. In 2019, the estimated region’s population was about 2.4 million [29]. The region has eight administrative districts; with an average population density of about 77 persons per square kilometre. Whereas the main activities of the population in rural areas are agriculture followed by livestock keeping, in urban centres there are large and small businesses.

### Study population

The study population included adults who usually reside (*de jure population*) in the selected households in Tanga city and Korogwe rural districts.

### Sample size estimation

We estimated a minimum sample size (N) of 795 adults from one urban district (Tanga city) and an equal size from one randomly selected rural district (Korogwe rural). The estimate is based on a 95% confidence level, 80% power of detecting differences in proportions of good knowledge and positive attitudes between the rural and urban populations. We applied a formula for comparison between two proportions [30]:

$$N=({Z_{\alpha }+Z_{\beta })}^{2}*\frac{p_{1}(1-p_{1})+p_{2}(1-p_{2})}{({p_{1}-p_{2})}^{2}}$$

in which Zα corresponds to the critical value of the normal distribution for a 95% confidence level (1.96), Z_β_ is the critical value of the normal distribution at 80% power of the study to detect the difference between the two proportions (0.84), p_1_ is the estimated proportion (44%) of rural adults with good knowledge about TB transmission [31]. The second proportion, p_2_ was used to estimate a similar proportion of adults dwelling in urban. Since there is no literature available for the urban population, it was estimated to be slightly higher (52%). Therefore, the estimated sample size for each of the two groups (rural and urban) was 636. We further adjusted for possible non-participation rate of 20%; yielding the final minimum sample of 795 adults per group.

### Sampling procedure

Since we aimed at comparing KAP among adults in urban and in rural settings, the sampling strategy was organized based on this structure. Although there are several towns in the region, most of them have a mixture of rural and urban (semi-urban) characteristics. There is only one city in the region, the Tanga city. Therefore, we purposefully selected Tanga City to represent the urban population. We used a four-stage sampling strategy to get the required study participants in this stratum. In the first stage, we randomly selected eight out of 24 wards in Tanga City. In the second, we randomly selected one street from each of the selected eight wards. The third stage involved a systematic selection of 100 households from each street, whereas the in the fourth stage we used a ballot box to randomly select one adult (at least 18 years old) for an interview.

In selecting adults to represent the rural population, we used a five-stage cluster sampling strategy. First, from a list of the available seven well-defined rural districts (Handeni, Kilindi, Korogwe, Lushoto, Mkinga, Muheza and Pangani), we randomly selected Korogwe rural district. Second, we randomly selected six wards out of the 20 wards in the district. The third stage involved, from each selected ward, we randomly selected one village. Fourth, in each village we systematically selected 130 households. Finally, in each selected household, we used a ballot box to randomly select only one adult for inclusion in the study.

### Data collection tools

We used an interview form to collect data on knowledge, attitudes and practices about TB infection control. The tool was designed based on expertise in the field and benchmarking with previous similar studies [21,32,33]. The interview form included structured questions organized into four main themes: (a) the background (social and demographic) information, (b) knowledge about TB infection, (c) practices about TB control and (d) attitudes towards TB infection. The tool was first prepared in English and translated to Kiswahili, a medium language of communication to almost everybody in Tanzania. However, in case of an adult proficient in English language, there were few surveys earmarked for this purpose.

The tool was pre-tested among adults in 10 households in one street and 10 others in one village. The village and the street were beside those earmarked for the main study. Based on the results from the pre-tests, we revised the tool to make sure the language is understandable and all options of possible answers for each item in the tool have been captured. Furthermore, through pre-testing we were able to assess the ability and skills of research assistants in collecting data. All research assistants had medical or social background and were well-trained to execute the task of data collection.

### Study variables and measures

#### Dependent variables

The main dependent variables were three: knowledge about TB infection, attitudes towards TB and practices about TB infection control.

#### Knowledge about TB infection

We asked nine questions about knowledge of TB symptoms; for example “what TB symptoms do you know?” There were eight questions on TB transmission; for example “How can an individual contract TB?” All questions were structured, pre-coded and mainly with the expected “yes”, “no” and “I don’t know” answers.

#### Attitudes towards TB infection

Participants self-reported their individual feelings about TB infection by answering nine questions, each on a Likert-scale with the expected opinions ranging from “Very strongly agree” to “Very strongly disagree”. These questions were towards both the disease and to the people with TB. Examples of these questions include, “People with TB should be avoided” and “People with TB should be feared”.

#### Practices about TB infection control

We asked study participants about what they were actually doing or they would do to protect themselves from contracting TB infection. Questions were about the general prevention practices and about health-seeking behaviour. For example, “How frequently do you do the following about TB: (a) boiling milk before use, (b) opening widows in your bed/living room”. Options were: (1) Always, (2) Most of the time, (3) Sometimes and (4) Not at all.

#### Independent variables

Independent variables were individual-level background characteristics: sex, age, education, marital status and the main source of income of the study participants.

### Data processing and analysis

Collected data were entered into statistical software (Statistical Package for Social Sciences; version 24) for processing and analysis. Responses on knowledge about TB infection were all aligned in a positive direction making sure that the correct answer is scored 1 and the wrong answer scored 0. Responses from questions for each component of knowledge (symptoms and transmission) were summed up to create a separate composite quantitative variable having possible score ranging positively from 0 to 9 and from 0 to 8 respectively. Later on, the overall knowledge score was created by a combination of knowledge on symptoms and of transmission scores, potentially ranging from 0 to 17. Similarly, responses about attitude towards TB infection were arranged from 1 = low score that implied low attitudes to 5 = high score implying positive attitude. In this study, a person having positive attitudes towards people with TB would mean an individual acknowledges TB exist, recognizes its impact on health and would seek or advise TB-infected individuals to seek the correct remedies.

During data processing, in case of questions with opposite direction, we reversed the coding. For each respondent we created a composite variable about attitudes with possible scores ranging from 9 to 45. Scores for practices ranged between 0 and 8. For each construct, we categorized a study participant with a score below 60% of the possible maximum score having a poor knowledge, negative attitudes or bad practices. The 60% mark was based on a modified Bloom’s cut-off and has been recently used in other studies of similar settings [34,35].

We calculated the means with standard deviations for quantitative variables and ran frequencies for categorical variables to get proportions. We applied Chi-squared test (χ^2^) to assess the association between a pair of categorical variables. In the multivariable analyses, we used multiple linear regression analysis to model overall scores of knowledge, attitudes and practices with the independent individual-level variables. Linear regression analysis was considered the best procedure in order to maintain the original measurements. Otherwise, the binary logistic regression analyses would have over-estimated the measures of association because all outcomes were not rare. In addition, the same procedure has been used previously in a KAP study in South Africa [25]. We calculated 95% confidence intervals (CI) as a measure of strength for the association. During the bivariate and multivariable analyses, the level of significance was set at 5%. Furthermore, we applied robust estimation of the standard errors and confidence intervals using bootstrapping method because of using cluster sampling rather than the simple random sampling technique.

### Ethics statement

The protocol of this research was reviewed and approved by the Muhimbili University of Health and Allied Sciences (MUHAS) Institutional Review Board. The Regional Administrative Secretary and the District Administrative Secretaries for Tanga City and Korogwe districts granted permission to conduct the survey in the study areas. While all literate study participants provided written informed consent, illiterate individuals provided the verbal. Thereafter, to document their consent, the literature and illiterate study participants signed or provided a thump-print respectively on their consent forms. Throughout the study, we adhered to anonymity, privacy, confidentiality and collected only information related to the study. Filled-in interview forms were stored in a safe locker with key only accessible by the principal investigator. Data in electronic form were password-protected and were only accessed by the authorized persons.

## Results

### Description of the study participants

We recruited 761 (95.7% recruitment rate) and 758 (95.3% recruitment rate) from the urban (Tanga city) and rural areas (Korogwe rural district) respectively. In Table 1, we present the distribution of the study participants by the study area and by background characteristics. The majority, 999 (65.8%), were females who also dominated in each of the two study areas. The median age of all study participants was 32.0 (Inter-quartile range = 19.0) years and did not differ by residence status (rural or urban). Regardless of residence, the majority 645 (84.8%) and 412 (54.3%) of the study participants from urban and rural areas respectively, reported their highest education level was primary school. This level was reported in urban areas, 645 (84.8%), and rural areas, 412 (54.4%). Almost half, 726 (47.8%) of all study participants reported business being their main source of income. The majority, 349 (46.0%) of study participants from rural areas reported peasantry and livestock keeping being their main sources of income.

**Table 1:** Distribution of study participants by background characteristics and place of residence.

Characteristics	Urban (n = 761)	Rural (n = 758)	Total (n = 1519)
	n (%)	n (%)	n (%)
**Sex**			
** Male**	250 (32.9)	270 (35.6)	520 (34.2)
** Female**	511 (67.1)	488 (64.4)	999 (65.8)
**Age group (years)** ^a^			
** 18–24**	155 (20.3)	171 (22.5)	326 (21.4)
** 25–44**	410 (53.9)	399 (52.6)	809 (53.4)
** 45–84**	143 (18.8)	144 (19.1)	287 (18.9)
**Highest education level**			
** Never or informal**	32 (4.2)	50 (6.6)	82 (5.4)
** Primary**	645 (84.8)	412 (54.4)	1057 (69.6)
** Secondary**	43 (5.7)	260 (34.3)	303 (19.9)
** Above secondary**	41 (5.4)	36 (4.7)	77 (5.1)
**Current marital status**			
** Married or cohabiting**	510 (67.0)	464 (61.2)	974 (69.1)
** Not married, widow, divorced**	251 (33.0)	294 (38.8)	545 (30.9)
**Main source of income**			
** Peasant or livestock keeping**	90 (11.8)	349 (46.0)	439 (28.9)
** Business**	451 (59.3)	275 (36.3)	726 (47.8)
** Salaried**	46 (6.0)	27 (3.6)	73 (4.8)
** Unemployed**	174 (22.9)	107 (14.1)	281 (18.5)

^a^Totals do not add up to 761 and 758 for urban and rural respectively due to missing data.

### Awareness of tuberculosis (TB)

In total, 1444 (95.1%) of the study participants reported being aware of TB. The proportions of study participants reporting aware of TB did not differ by the study area, 717 (94.2%) [95%CI = 92.3, 95.7] and 727 (95.9%) [95%CI = 94.2, 97.2] for urban and rural areas respectively.

### Overall knowledge about TB infection

The average score for the overall knowledge among all study participants was 7.2 (standard deviation (SD) = 2.9). Of the 1359 study participants who responded to all items in this construct, 601 (44.2%) had good overall knowledge. While 460 (64.2%) study participants from urban areas had good overall knowledge, less than a quarter, 141 (22.0%) from rural settings had good overall knowledge about TB infection (χ^2^ = 244.5, *p*< 0.001) (Table 2).

**Table 2:** Association between residence and knowledge, attitudes and practices regarding tuberculosis.

Knowledge, attitudes and practices	Urban (n = 717)	Rural (n = 727)	χ^2^	*p*-value
	n (%)	n (%)		
**Good overall knowledge**	460 (64.2)	141 (22.0)^a^	244.5	< 0.001
**Knowledge on symptoms**	660 (92.0)	551 (75.8)	70.5	< 0.001
**Knowledge on transmission**	678 (94.7)	274 (42.7)	436.9	< 0.001
**Positive attitude**	113 (15.8)	45 (6.2)	33.9	< 0.001
**Good practices**	692 (96.5)	93 (56.4)^a^	220.9.4	< 0.001

^a^Data were available for only 165 study participants.

### Knowledge about TB symptoms

The average score on knowledge about TB symptoms among all study participants was 2.7 (SD = 2.3) and it was significantly higher among study participants from urban than rural settings, 3.2 (SD = 2.6) and 2.2 (SD = 1.6) respectively (*p*<0.001). In total, 1211 (83.9%) had good knowledge about TB symptoms. Knowledge of TB symptoms was dependent on residence such that 660 (92.0%) as compared to 551 (75.8%) of the study participants from urban and rural areas respectively had good knowledge on TB symptoms (χ^2^ = 70.5, *p*< 0.001) (Table 2).

### Knowledge about TB transmission

The average score for knowledge about TB transmission among all study participants was 4.5 (SD = 1.5). Of the 1358 study participants who respondent to all items, 952 (70.1%) had good knowledge about TB transmission. While 678 (94.7%) of the study participants residing in urban areas had good knowledge about TB transmission, only 274 (42.7%) from rural areas had such knowledge (χ^2^ = 436.9, *p*< 0.001) (Table 2).

### Attitudes about TB infection

When assessing the strength of consistency of the nine items we used to measure attitudes on a Likert-scale, *Cronbach’s alpha* (α) was 0.742; indicating a good reliability. The overall average attitudes score about TB infection was 19.1 (SD = 6.2). The average score was significantly (*p*<0.001) higher, 22.7 (SD = 4.0) among adults in urban areas as compared to their counterparts, 15.6 (6.0). In general, of the 1444 study participants, 158 (10.9%) had positive attitudes towards TB infection. There were significantly more study participants from urban areas, 113 (15.8%) with positive attitudes towards TB infection as compared to adults dwelling in the rural settings, 45 (6.2%), (χ^2^ = 33.9, *p*< 0.001) (Table 2).

### Practices about TB infection control

Among 882 study participants who respondent to this construct, 785 (89.0%) had good practices in controlling TB infection. Their average score on this construct was 5.0 (SD = 1.2). Some of the unexpected practices reported controlling TB infection included, avoiding hand shake 734 (83.2%), having good diet 799 (90.6%) and avoiding sharing utensils 576 (65.3%). The average score about practices in controlling TB infection was higher, 5.1 (SD = 0.9) among adults in urban as compared to 4.0 (SD = 1.7) for those in rural areas (*p*<0.001). Significantly more adults, 692 (96.5%) from urban areas as compared to 93 (56.4%) of the rural adult population had good practices about TB infection control (χ^2^ = 220.9, *p*<0.001) (Table 2).

### Factors associated with overall knowledge scores about TB infection control

In a multiple linear regression analysis for the overall knowledge scores about TB infection on individual-level factors, regardless of place of residence (whether rural or urban), adults with secondary education were significant positively associated with higher overall knowledge scores about TB infection. Adjusting for residence, they had almost 2 scores of overall knowledge higher (beta-coefficient (β) = 1.9, 95%CI = 1.0, 2.8; *p* = 0.001) than adults reporting never been to school or with informal education (Table 3).

**Table 3:** Association of individual-level factors and overall knowledge scores on TB infection.

Variable	β-coefficient^a^	SE^b^	95%CI^b^	*p-*value
**Sex**				
** Male**	Reference	―	―	―
** Female**	0.0	0.158	-0.3, 0.3	0.994
**Age** ^c^				
** Years**	0.0	0.005	0.0, 0.0	0.909
**Highest education level**				
** Never of informal**	Reference	―	―	―
** Primary**	0.4	0.328	-0.2, 1.1	0.195
** Secondary**	1.9	0.454	1.0, 2.8	0.001
** Above secondary**	0.9	0.564	-0.1, 2.2	0.096
**Current marital status**				
**In union**^d^	0.2	0.150	-0.1, 2.2	0.273
**Not in union**^e^	Reference	―	―	―
**Main source of income**				
** Peasant/livestock keeping**	0.3	0.312	-0.4, 0.9	0.367
** Business**	0.2	0.176	-0.2, 0.5	0.344
** Salaried**	-0.1	0.420	-1.0, 0.7	0.819
** Unemployed/other**	Reference	―	―	―

^a^Adjusted for residence (rural and urban).

^b^With bootstrapping adjustment.

^c^Age was treated a continuous variable in the model.

^d^Married or cohabiting.

^e^Never or previously married.

#### Factors associated with attitudes TB infection

Contrary to the overall knowledge scores, attitudes towards TB infection control was negatively associated with the education status of the adults. Irrespective of place of residence, adults with secondary had 5.2 significantly less scores of having negative attitudes as compared to those never been to school or with informal education (β = -5.2, 95%CI = -7.2, -3.4; *p* = 0.001). Likewise, adults with higher than secondary education had 4.3 less scores of having negative attitudes as compared to those never been to school or with informal education (β = -4.3, 95%CI = -6.8, -1.9; *p* = 0.001) (Table 4).

**Table 4:** Association between individual-level factors and attitudes towards TB infection.

Variable	β-coefficient^a^	SE^b^	95%CI^b^	*p-*value
**Sex**				
** Male**	Reference	―	―	―
** Female**	0.4	0.333	-0.3, 1.0	0.278
**Age** ^c^				
** Years**	0.0	0.011	0.0, 0.0	0.423
**Highest education level**				
** Never of informal**	Reference	―	―	―
** Primary**	-0.7	0.930	-2.6, 1.0	0.460
** Secondary**	-5.2	0.986	-7.2, -3.4	0.001
** Above secondary**	-4.3	0.228	-6.8, -1.9	0.001
**Current marital status**				
**In union**^d^	-0.1	0.334	-0.7, 0.6	0.897
**Not in union**^e^	Reference	―	―	―
**Main source of income**				
** Peasant/livestock keeping**	-2.6	0.461	-3.5, -1.7	0.001
** Business**	0.0	0.414	-0.8, 0.8	0.997
** Salaried**	2.1	0.914	0.2, 3.9	0.017
** Unemployed/other**	Reference	―	―	―

^a^Adjusted for residence (rural and urban).

^b^With bootstrapping adjustment.

^c^Age was treated a continuous variable in the model.

^d^Married or cohabiting.

^e^Never or previously married.

While peasants and livestock keepers were negatively associated with positive attitudes towards TB infection control, adults working for salaries were positively associated with positive attitudes towards TB infection control. Peasant and livestock keepers had 2.6 less of having negative attitudes as compared to unemployed adults (β = -2.6, 95%CI = -3.5, -1.7; *p* = 0.001). Salaried adults were positively associated with attitudes towards TB infection control such that they had 2.1 higher scores of attitudes as compared to unemployed adults (β = 2.1, 95%CI = 0.2, 3.9; *p* = 0.017).

#### Factors associated with practices towards TB control

In Table 5, we present multiple linear regression analysis outcomes of scores of practices towards TB prevention and control on selected individual-level variables. Education was negatively associated with scores of practices towards TB control. An adult with primary education had 0.6 less scores of practices towards TB preventive control measures as compared to adults who have never been to school or those with informal education, (β = -0.6, 95%CI = -1.0, -0.3; *p* = 0.001). Similarly, controlling for residence, adults with secondary education had 1.4 less scores of practices towards TB control as compared to those never been in school or having informal education (β = -1.4, 95%CI = -1.9, -1.0; *p* = 0.001).

**Table 5:** Association between individual-level factors and practices towards TB infection control.

Variable	β-coefficient^a^	SE^b^	95%CI^b^	*p-*value
**Sex**				
** Male**	Reference	―	―	―
** Female**	0.0	0.086	-0.2, 0.2	0.980
**Age** ^c^				
** Years**	0.0	0.002	-0.1, 0.0	0.230
**Highest education level**				
** Never of informal**	Reference	―	―	―
** Primary**	-0.6	0.160	-1.0, -0.3	0.001
** Secondary**	-1.4	0.215	-1.9, -1.0	0.001
** Above secondary**	-0.8	0.290	-1.4, -0.3	0.003
**Current marital status**				
**In union**^d^	-0.1	0.080	-0.2, 0.1	0.354
**Not in union**^e^	Reference	―	―	―
**Main source of income**				
** Peasant/livestock keeping**	-0.4	0.135	-0.7, -0.1	0.010
** Business**	-0.1	0.087	-0.2, 0.1	0.515
** Salaried**	0.3	0.241	-0.2, 0.8	0.232
** Unemployed/other**	Reference	―	―	―

^a^Adjusted for residence (rural and urban).

^b^With bootstrapping adjustment.

^c^Age was treated a continuous variable in the model.

^d^Married or cohabiting.

^e^Never or previously married.

In addition, peasants and respondents engaging in livestock keeping were significant negatively associated with scores of practices towards TB infection control (β = -0.4, 95%CI = -0.7, -0.1; *p* = 0.010) (Table 5).

## Discussion

In this study, we assessed knowledge, attitudes and practices towards prevention and control measures against TB infection among rural and urban adult populations in northeast Tanzania. Although the majority (95%) of adults from the study area are aware of TB, less than half (44%) has good overall knowledge about TB infection. In this study, the proportion of the study participants with good overall knowledge is almost the same as that of Nigeria but lower than in the Gambian study [26,27]. The variation in the proportions of population with good knowledge about TB infection between urban and rural in not a new phenomenon. For example, a study in Lesotho [20], Ethiopia [22] and Nigeria [26] have also reported differences of TB knowledge between urban and rural communities. One possible explanation for the underlying source of variability in the good overall knowledge between rural and urban could be the education status that favors most of the population in urban area [36].

Despite a larger proportion of adults having good knowledge on symptoms, transmission and good practices about TB infection, it is surprising that as few as only 11% have positive attitudes towards the TB infection. This level is in contrast with recent figures from Nigeria, Gambia and Ethiopia [26,27,37]. In 2019, a study in Nigeria reported a link between education among men and their families and TB knowledge scores [38]. Furthermore, Balogun, et al., report the association between knowledge and attitudes in Nigeria [24]. Nevertheless, negative attitudes will always imply poor health seeking behaviours and adherence to medications.

It is of critical importance to underscore the knowledge gap especially of TB transmission in rural areas in this study. It is underscored that inadequate knowledge about TB symptoms and transmission is among the threats to employ good prevention practices [24,27,31,39]. Therefore, one of the implications from this study is that, with poor knowledge and negative attitudes towards TB it is likely the community will have poor efforts in prevention practices against TB infection and possibly end up with limited health care seeking behaviors [40,41].

In this study, there is a negative association between practice and attitudes towards TB infection control and adults working as peasants or livestock keepers. Although the negative attitudes about TB has also been reported in Ethiopia more among livestock keepers than the sedentary communities [38], the mechanism through which both negative attitudes and practice evolve are unclear. However, in the same study in Ethiopia, they mention the observed high overall perceived stigma could be contributing to a negative attitudes [38].

It is imperative to note that more than 70% of the population in Tanzania resides in rural areas [42]. Having a substantial variability between rural and urban communities with respect to knowledge, attitudes and practices towards prevention and control of TB infection favoring the urban population, suggests for strategies to find ways of imparting TB knowledge on symptoms and transmission specifically to the rural population as long as this phenomenon is consistent even in other areas.

There are variations of KAP definitions in different health-related behaviours between and within countries. Therefore, even after benchmarking our tool against other similar studies at national and international levels and the further validation in terms of its face and content, the differences we are observing in this study could be a result of the variability in KAP definition.

Our study has several potential limitations. One, although we maximally trained our research assistants, who had also medical background, we cannot rule out the possibility of social desirability bias. Two, due to the literacy level among our study participants especially in rural areas, we decided to use a face-to-face interview schedule. In spite of its merits, one of the disadvantages of this survey method is the possibility of having the interviewer effects leading to the interviewer bias. The extensive training of the research assistants could have reduced this bias.

## Conclusions

In conclusion, TB awareness among adults in rural and urban areas of northeast Tanzania is very high. Adults have inadequate overall knowledge and extremely negative attitudes towards TB. The most disadvantaged adults are those residing in rural than their counterparts in urban settings. Factors that were strongly associated with scores on knowledge, attitudes and practices included education (primary, secondary and above secondary) and the main source of income (peasantry/livestock keeping and salaried). Findings from this study suggest the need to have community-health campaigns especially among rural communities. In order to improve community members’ knowledge, attitudes and practices towards the prevention and control of TB in Tanzania, we recommend focused community-based campaigns to educate the rural and urban communities on several aspects of TB. Furthermore, a comprehensive health education strategy is needed to patients and their supporters visiting the health facilities and outreach stations. We recommend for further research to examine possible sources of the negative association between education status and scores of attitude and practices on TB infection control.

## Supporting information

S1 File TB Questionnaire English. Questionnaire in English version.
(DOCX)

S2 File TB Questionnaire Kiswahili. Questionnaire in Kiswahili version.
(DOCX)

S3 File TB Tanzania data. Data file in SPSS data format.
(SAV)

## Abbreviations

CI: Confidence interval
HIV&AIDS: Human immunodeficiency virus infection and Acquired Immunodeficiency Syndrome
KAP: Knowledge, attitudes and practices
SE: Standard error
TB: Tuberculosis
WHO: World Health Organization
