# Supplementary material for: Knowledge, attitudes and practices on tuberculosis infection prevention and associated factors among rural and urban adults in northeast Tanzania: A cross-sectional study
Source: PLOS Glob Public Health. 2021 Dec 8;1(12):e0000104. doi: 10.1371/journal.pgph.0000104 (PMC10022383; doi:10.1371/journal.pgph.0000104)
Supplement: S1 File — Questionnaire in English version. (DOCX) [file pgph.0000104.s001.docx]

S1 File. TB QUESTIONNAIRE_ENGLIS. Questionnaire in English version.

**KNOWLEDGE, ATTITUDES, PERCEPTION AND PRACTICES TOWARDS TUBERCULOSIS IN TANGA, TANZANIA**

| **QCODE** | **QUESTION** |  |
| --- | --- | --- |
| **A1** | Identification number | [..................................] |
| **AA** | Name/code of interviewer | ........................................................ |
| **AINT** | Date of interview | ........./............/.................. |
| **A2** | District | 1. Korogwe 2. Tanga City |

**A. Socio-demographic information**

| **QCODE** | **QUESTIONS** | **RESPONSES** |
| --- | --- | --- |
| **A2S** | Name of the street |  |
| **A2W** | Name of the ward | ......................................................................... |
| **A3** | Sex of the respondent | Male...............1 Female..........................2 |
| **A4** | How old are you? | Years …............................. |
| **A44** | What is your relationship with the head of the household | I am the head of the household....1  Wife.............................................2  Child............................................3  Brother/Sister...............................4  Other............................................5 |
| **A5** | What is your current marital status? | Married.........................................1  Single ...........................................2  Divorced .......................................3  Cohabiting ....................................4  Widow ..........................................5 |
| **A6** | What is the highest level of education that you achieved? | Never in the school...................................1  Incomplete primary school.......................2  Complete primary school.........................3  Secondary school.....................................4  Certificate/Diploma.................................5  Madrassa/Religious education.................6  Adult education…………………..…….7 |
| **A7** | What do you do for living?  (ONLY ONE RESPONSE) | Business.............................................1  Agriculture.........................................2  Keeping animals.................................3  Formal employment (salaried) ..........4  Other (mention)..................................5 |
| **AD** | How long would it take you to reach the nearest health facility on foot? | Hours…………  Minutes……… |
| **APU**  **APA** | How many people normally live in this household? | Under-fives 5……………………..  Five years and above…………….. |

**S ECTION B: PRACTICES TOWARDS TB PREVENTION**

| **QCODE** | **QUESTION** | **RESPONSES** |
| --- | --- | --- |
| **A8** | Have you ever heard anything about tuberculosis (TB)? | Yes ..........................1  No ......................2 (THANK THEM AND CLOSE THE INTERVIEW) |

How frequently do you do the following about TB?

| **B1** | Boiling milk before use | 1. Always  2. Most of the time  3. Sometimes  4. Not at all |
| --- | --- | --- |
| **B2** | Hand-shaking with anybody suspecting a TB case | 1. Always  2. Most of the time  3. Sometimes  4. Not at all |
| **B3** | Put on a mask when suspecting TB | 1. Always  2. Most of the time  3. Sometimes  4. Not at all |
| **B4** | Sharing of utensils | 1. Always  2. Most of the time  3. Sometimes  4. Not at all |
| **B5** | Avoid contacts in public buildings | 1. Always  2. Most of the time  3. Sometimes  4. Not at all |
| **B6** | Opening widows in your bed/living room | 1. Always  2. Most of the time  3. Sometimes  4. Not at all |
| **B7** | Have a good diet especially when suspecting TB symptoms | 1. Always  2. Most of the time  3. Sometimes  4. Not at all |
| **B8** | Visit health facility when having TB symptoms | 1. Always  2. Most of the time  3. Sometimes  4. Not at all |

**SECTION C: KNOWLEGDE ABOUT TB INFECTION**

| **QCODE** | **QUESTION** | **RESPONSES** | | |
| --- | --- | --- | --- | --- |
| **C1** | Has anybody in this household experienced any of the following diseases?  (Read loud each option) | Malaria…… …..1  Dysentery… ….2  Pneumonia… …3  HIV/AIDS……..4  TB…………..….5  Corona…………6  Vomiting……….7  BP…………..…..8  Kwashiorkor……9  Diabetes……….10  Other…………………………….11 | | |
| **C2** | From which source did you FIRST hear about TB?  **DO NOT READ ALOUD THE ANSWERS (Only one response)** | 1. Radio....................................... 2. Newspapers................................ 3. TV.......... 4. Friends/Relatives.......................... 5. Health campaigns........................ 6. Workshop..................................... 7. Posters................................. 8. Community gatherings..................... 9. Health workers............. 10. Social media 11. Education institutions 12. Other (mention)................... | Yes | No |
|  |  |  | 1  1  1  1  1  1  1  1  1 | 2  2  2  2  2  2  2  2  2 |
| **C3** | When was the last time you heard about TB? (Do NOT read out**)** | Less than a month ago………1  About one month....................2  More than a month………......3  Do not remember.....................4 | | |

**SECTION D: KNOWLEDGE ABOUT TB PREVENTION**

| **QCODE** | **QUESTION** | **RESPONSES** | | |
| --- | --- | --- | --- | --- |
| **D10** | What organisms that cause pulmonary TB? | 1. Virus.............................................. 2. Bacteria.............................................. 3. Other.................................... 4. Don’t know..................................... | Yes  1  1  1  1 | No  2  2  2  2 |
| **D11** | How can an individual contract TB?  **( Circle all that apply)**  **DO NOT READ ALOUD** | 1. Hand shake…………………….. 2. Smoking 3. Sharing utensils 4. Drink fresh milk without boiling........ 5. Contact handles in public places (door handles, hand hangers in the buses, etc…………… 6. Sexual intercourse…………………………… 7. From mother to unborn child………………… 8. Through air transmission from an infected person (cough or sneezing……………………….. 9. Don’t know..................................................... 10. Other (mention).................................................. | Yes  1  1  1  1  1  1  1  1  1  1  1 | No  2  2  2  2  2  2  2  2  2  2  2 |
| **D12** | What TB symptoms do you know? (Circle all that apply**)**    **DO NOT READ ALOUD** | 1. Loss of appetite...................................................... 2. Unexplained fevers of more than seven days.............. 3. Chest pains……… 4. Prolonged cough of more than two weeks… 5. Difficulty in breathing ………………… 6. Night sweats............................ 7. Weight loss................................................... 8. Coughing some blood.......................................... 9. Constant fatigue........................................................ 10. Don’t know........................................................ 11. Other (mention)................................................. | Yes | No |
|  |  |  | 1  1  1  1  1  1  1  1  1  1  1 | 2  2  2  2  2  2  2  2  2  2  2 |
| **D13** | How can you protect yourself against TB?(Circle all that apply)  **DO NOT READ ALOUD** | 1. Boil fresh milk...................................... 2. Avoid hand shaking............................... 3. Kufunika mdomo na pua wakati wa kukohoa au kupiga chafya............................................. 4. Avoid sharing utensils.............................. 5. Sanitize when in contact with public handles (in busses, elevators, door-handles, etc..................... 6. Close doors and windows........................ 7. Good diet..................................... 8. Prayers....................................................... 9. Don’t know……………………………… 10. Other (mention)................................................... 11. Avoid crowding……………………. | Yes  1  1  1  1  1  1  1  1  1  1  1 | No  2  2  2  2  2  2  2  2  2  2  2 |
| **D14** | How expensive is the cost of TB examination and treatment in Tanzania?  (Circle only one option)  **DO NOT READ ALOUD** | Free/No cost……………………………..1  Normal, acceptable………………………2  Somehow expensive………….………….3  Very expensive………………………….4  Don’t know……………………………….5  Interviewer: If amount is mentioned, record it here……………. | | |

**SECTION E: RELATED ATTITUDES TOWARDS TB**

**[I will read some statements about TB, please tell me if you strongly agree or disagree for each sentence]**

| **QCODE** | **Statement** | **Strongly disagree** | **Disagree** | **Neither agree, nor disagree** | **Agree** | **Strongly agree** |
| --- | --- | --- | --- | --- | --- | --- |
| **E1** | TB is a punishment from God | 1 | 2 | 3 | 4 | 5 |
| **E2** | TB is due to witchcraft | 1 | 2 | 3 | 4 | 5 |
| **E3** | TB is due to dry period with strong sun | 1 | 2 | 3 | 4 | 5 |
| **E4** | TB is curable | 1 | 2 | 3 | 4 | 5 |
| **E5** | TB has been imposed by Whites to kill Africans | 1 | 2 | 3 | 4 | 5 |
| **E6** | HIV/AIDS is due to TB | 1 | 2 | 3 | 4 | 5 |
| **E7** | TB is due to HIV/AIDS | 1 | 2 | 3 | 4 | 5 |
| **E8** | People with TB should be feared | 1 | 2 | 3 | 4 | 5 |
| **E9** | People with TB should be avoided | 1 | 2 | 3 | 4 | 5 |

| **SECTION F: SOURCES OF INFORMATION** | | |
| --- | --- | --- |
| **F1** | Do you think you have enough information about TB | Yes…..................1  No………………2  Don’t know …….3 |
| **F2** | Would you like to have additional information about TB? | Yes….......1  No……….(Go to F**4**) |
| **F3** | If YES, what kind of information would you like to get? | Symptoms…………….…….1  Modes of transmission…...…2  Treatment……………………3  Prevention……………………4  Other (mention)……………...5 |
| **F4** | What do you think are the best sources of additional information about TB you and your friends should get?  (CIRCLE ALL THAT APPLY**)** | Newspapers/Magazines…………..1  Radio ……………………………..2  TV………………………..……….3  Posters……………………………..4  Leaflets/other publications …………5  Health care providers……………….6  Relatives/Friends/Neighbors………7  Religious/Community leaders………8  Teachers……………………………..9  Other (Mention)…………………….10 |
| **F5** | What kind of worries do you get when you think about TB .......................................................... | |
