# Supplementary material for: Knowledge, attitudes and practices on tuberculosis infection prevention and associated factors among rural and urban adults in northeast Tanzania: A cross-sectional study
Source: PLOS Glob Public Health. 2021 Dec 8;1(12):e0000104. doi: 10.1371/journal.pgph.0000104 (PMC10022383; doi:10.1371/journal.pgph.0000104)
Supplement: S2 File — Questionnaire in Kiswahili version. (DOCX) [file pgph.0000104.s002.docx]

S2 File. TB QUESTIONNAIRE_KISWAHILI. Questionnaire in English version.

**MAARIFA/UELEWA, MTAZAMO NA MATENDO KUHUSU UGONJWA WA KIFUA KIKUU, TANGA TANZANIA**

| **QCODE** | **SWALI** |  |
| --- | --- | --- |
| **A1** | Namba ya utambulisho ya dodoso | [..................................] |
| **AA** | Jina la mhojaji | ........................................................ |
| **AINT** | Tarehe ya mahojiano | ........./............/.................. |
| **A2** | Wilaya | 1. Korogwe 2. Tanga jijini |

**A. Taarifa binafsi/ki-demografia**

| **QCODE** | **SWALI** | **MAJIBU** |
| --- | --- | --- |
| **A2S** | Jina la Mtaa |  |
| **A2W** | Jina la Kata | ......................................................................... |
| **A3** | Jinsi | Mwanaume...............1 Mwanamke..........................2 |
| **A4** | Una umri gani? | Miaka …............................. |
| **A44** | Uhusiano na mkuu wa kaya | Mkuu wa kaya mwenyewe.............1  Mke.................................................2  Mtoto..............................................3  Kaka/Dada......................................4  Mwingine.......................................5 |
| **A5** | Hali ya ndoa | Ameoa/olewa..........................................................1  Hajaoa/olewa .........................................................2  Tengana/talikiwa ...................................................3  Anaishi kinyumba .................................................4  Mjane/mgane .........................................................5 |
| **A6** | Kiwango chako cha juu cha elimu? | Hajaenda shule/hakusoma............................................1  Hakumaliza elimu ya msingi.......................................2.  Amemaliza elimu ya msingi........................................3  Sekondari.....................................................................4  Stashahada/shahada/astashahada.................................5  Madrasa/Elimu ya dini................................................6  Darasa la kusoma na kuandika/elimu ya watu wazima……………….7 |
| **A7** | Je, kipato chako kinatokana na haswa na shughuli ipi?  (JIBU MOJA) | Biashara.............................................1  Kilimo/mkulima................................2  Ufugaji..............................................3  Muajiriwa..........................................4  Nyingine(taja)....................................5 |
| **AD** | Unatumia/ungetumia muda gani kwenda kwa mguu kwenye kituo cha huduma za afya kilichopo karibu nawe? | Robo saa…………1  Zaidi ya robo saa mpaka nusu saa…2  Zaidi ya nusu saa mpaka saa moja………3 |
| **APU**  **APA** | Unaishi kwenye kaya ya watu wangapi? | Watoto umri chini ya miaka 5……………..  Watu wenye umri zaidi ya miaka 5…………….. |

**SEHEMU B: VITU UNAVYOFANYA KWA MAISHA YA KILA SIKU**

| **QCODE** | **SWALI** | **JIBU& ALAMA** |
| --- | --- | --- |
| **A8** | Je! Umewahi kusikia ugonjwa unaitwa kifua kikuu (TB)? | Ndio ..........................1  Hapana ......................2 (MSHUKURU, MALIZIA USAILI) |

Ni mara ngapi unafanya yafuatayo unapohisi ugonjwa wa TB?

| **B1** | Kuchemsha maziwa kabla ya kuyatumia | 1. Kila mara  2. Mara nyingi  3. Mara chache  4. Hata mara moja |
| --- | --- | --- |
| **B2** | Kusalimiana kwa kushikana mikono | 1. Kila mara  2. Mara nyingi  3. Mara chache  4. Hata mara moja |
| **B3** | Kuvaa barakoa | 1. Kila mara  2. Mara nyingi  3. Mara chache  4. Hata mara moja |
| **B4** | Kuchangia vyombo vya chakula | 1. Kila mara  2. Mara nyingi  3. Mara chache  4. Hata mara moja |
| **B5** | Kuacha kugusagusa sehemu mbaimbali maeneo ya jumuiya | 1. Kila mara  2. Mara nyingi  3. Mara chache  4. Hata mara moja |
| **B6** | Kufungua madirisha | 1. Kila mara  2. Mara nyingi  3. Mara chache  4. Hata mara moja |
| **B7** | Kuwa na lishe bora | 1. Kila mara  2. Mara nyingi  3. Mara chache  4. Hata mara moja |
| **B8** | Kwenda kwenye kituo cha afya | 1. Kila mara  2. Mara nyingi  3. Mara chache  4. Hata mara moja |

**SEHEMU C: MAARIFA/UELEWA KUHUSU TB**

| **QCODE** | **SWALI** | **JIBU& ALAMA** | | |
| --- | --- | --- | --- | --- |
| **C1** | Je, kwenye kaya hii, kuna mtu ameugua magonjwa yafuatayo?  (MSOMEE NA JINA KILA JIBU) | Malaria………..1  Kuharisha…….2  Nimonia………3  UKIMWI……..4  TB…………….5  Corona…………6  Kutapika……….7  Presha…………..8  Kwashiakor……9  Kisukari……….10  Mengineyo…………………………….11 | | |
| **C2** | Kwa mara ya kwanza, ulisikia wapi kuhusu kifua kikuu?  **USIMSOMEE (Jibu moja tu)** | 1. Redio....................................... 2. Gazeti................................. 3. Kipindi cha runinga.......... 4. Marafiki./ndugu............................. 5. Kampeni ya afya........................ 6. Mafunzo..................................... 7. Mabango................................. 8. Mikutano ya kijiji/jamii...................... 9. Wafanyakazi wa afya/mifugo............. 10. Mitandao ya kijamii 11. Nyingine(taja)................... | Ndio | /Hapan |
|  |  |  | 1  1  1  1  1  1  1  1  1 | 2  2  2  2  2  2  2  2  2 |
| **C3** | Ni lini ilikuwa mara ya mwisho kusikia ujumbe wowote kuhusu kifua kikuu? (**usitaje machaguo)** | Chini ya mwezi mmoja uliopita………1  Mwezi mmoja uliopita...........................2  Zaidi ya mwezi mmoja uliopita..........3  Sikumbuki.......................4 | | |

**SEHEMU D2: MAARIFA/UELEWA WA MAAMBUKIZI, DALILI NA JINSI YA KUJIKINGA NA TB**

| **QCODE** | **SWALI** | **JIBU** | | |
| --- | --- | --- | --- | --- |
| **D10** | Je, ni vimelea gani wanaosababisha ugonjwa wa kifua kikuu kwa binadamu?  **USIMSOMEE** | 1. Virusi.............................................. 2. Bakteria.............................................. 3. Nyingine.................................... 4. Sijui..................................... | Yes  1  1  1  1 | No  2  2  2  2 |
| **D11** | Mtu anawezaje kupata kifua kikuu?  **( Tafadhali zungushia majibu yote yaliyotajwa)**  **USIMSOMEE** | 1. Kwa kushikana mikono/kusalimiana…………….. 2. Kuvuta sigara……………………………………… 3. Kupitia kuchangia vyombo vya chakula………. 4. Kunywa maziwa ambayo hayajachemshwa........ 5. Kwa kushika vitu sehemu za umma (vitasa vya milango, vishikio kwenye vyombo vya usafiri na kadhalika…………… 6. Kwa njia ya ngono 7. Kupitia kwa mama kwenda kwa mtoto 8. Kupitia hewa pale ambapo mtu mwenye kifua kikuu akikohoa au kupiga chafya……………………….. 9. Sijui..................................................... 10. Nyingine(taja).................................................. | 1  1  1  1  1  1  1  1  1  1  1 | 2  2  2  2  2  2  2  2  2  2  2 |
| **D12** | Je, ni dalili gani za kifua kikuu unazo zifahamu? (**Tafadhali zungushia majibu yote yaliyotajwa)**    **USIMSOMEE** | 1. Kukosa hamu ya kula……….. 2. Homa bila sababu ambayo inayodumu kwa zaidi ya siku saba........................................................................... 3. Maumivu ya kifua 4. Kikohozi kinachodumu zaidi ya wiki mbili… 5. Kupumua kwa shida............................. 6. Kukohoa damu.......................................... 7. Kupungua uzito.................................................... 8. Kikohozi mchanganyiko na damu 9. Uchovu usioisha………………………………. 10. Sijui........................................................ 11. Nyingine(taja)................................................. | Ndio | Hapana |
|  |  |  | 1  1  1  1  1  1  1  1  1  1  1 | 2  2  2  2  2  2  2  2  2  2  2 |
| **D13** | Je mtuanawezaje kujikinga na ugonjwa wa kifua kikuu?(**Tafadhali zungushia majibu yote yaliyotajwa**)  **USIMSOMEE MACHAGUO** | 1. Kuchemsha maziwa...................................... 2. Kuepuka kushikana mikono............................... 3. Kufunika mdomo na pua wakati wa kukohoa au kupiga chafya............................................. 4. Kuepuka kuchangia vyombo.............................. 5. Kuosha/ kunawa mikono unaposhika vitu kwenye maeneo ya umma.............................. 6. Kufunga madirisha nyumbani........................ 7. Kupata lishe bora..................................... 8. Kwa kusali....................................................... 9. Sijui……………………………… 10. Nyingine(taja)................................................... 11. Kuepuka misongamano……………………. | Ndio | Hapana |
|  |  |  | 1  1  1  1  1  1  1  1  1  1  1 | 2  2  2  2  2  2  2  2  2  2  2 |
| **D14** | Je, unafahamu gharama za uchunguzi na matibabu ya kifua kikuu zikoje nchini hapa?  (Chagua jibu moja)  **USIMSOMEE MAJIBU** | Ni bure/ hakuna malipo…………………..1  Bei ya kawaida……………………………2  Ni kwa kiasi flani/gharama kiasi………….3  Gharama sana…………………………….4  Sijui……………………………………….5  Muhojaji: Kama mhojiwa akitaja kiasi cha pesa, andika kiasi hapa……………. | | |

**SEHEMU E: [Sasa nitakusomea sentensi kuhusiana na masuala mbalimbali yahusuyo kifua kikuu (TB), tafadhali niambie unakubaliana au haukubaliani kwa kiasi gani kwa kila sentensi.**

| **QCODE** | **KAULI** | **Sikubaliani kabisa** | **sikubaliani** | **Sina hakika** | **nakubaliana** | **nakubalianakabisa** |
| --- | --- | --- | --- | --- | --- | --- |
| **E1** | Kifua kikuu ni ugonjwa hatari sana | 1 | 2 | 3 | 4 | 5 |
| **E2** | Kifua kikuu ni tishio kwa ustawi wa jamii na uchumi wa jamii hii | 1 | 2 | 3 | 4 | 5 |
| **E3** | Unafkiri upo katika hatari ya kupata ugonjwa wa kifua kikuu | 1 | 2 | 3 | 4 | 5 |
| **E4** | Kifua kikuu ni ugonjwa sugu | 1 | 2 | 3 | 4 | 5 |
| **E5** | Kifua kikuu ni ugonjwa wa watu masikini/hali ya chini | 1 | 2 | 3 | 4 | 5 |
| **E6** | Inawezekana kudhibiti maambukizi/kuenea kwa kifua kikuu katika jamii | 1 | 2 | 3 | 4 | 5 |
| **E7** | Vituo vya kutolea huduma za afya kwenye jamii hii vimejitosheleza kushughulikia /kutoa huduma za kifua kikuu | 1 | 2 | 3 | 4 | 5 |
| **E8** | Watu wengi katika jamii hii wana huruma au hamasa ya kumsaidia mtu ambae ana kifua kikuu? | 1 | 2 | 3 | 4 | 5 |
| **E9** | Wewe binafsi unahisi huruma au hamasa ya kumsaidia mtu mwenye kifua kikuu | 1 | 2 | 3 | 4 | 5 |

| **SEHEMU G: VYANZO VYA HABARI** | | |
| --- | --- | --- |
| **F1** | Je! Unadhani kwamba una taarifa za kutosha kuhusu ugonjwa wa kifua kikuu? | ndiyo….......1  hapana…….2  sijui |
| **F2** | Je unatamani ungepata maelezo zaidi kuhusu kifua kikuu? | ndiyo….......1  hapana------2 (**Nenda F4**) |
| **F3** | Kama NDIYO, ni kitu gani ungependa kufahamu Zaidi kuhusu kifua kikuu? | Dalili…………………….1  Jinsi/namnayakuambukiza………………….2  Matibabu……………………………………3  Kujikinga……………………………………4  Nyingine (taja)………………5 |
| **F4** | Je! Ungependeza vyanzo gani/vipi vya habari/taarifa vinaweza kuwafikia zaidi watu kama wewe ili kupata taarifa zaidi kuhusu kifua kikuu?  (ZUNGUSHIA KILA JIBU ALILOLITAJA**)** | Gazeti/ Jarida……………………..1  Redio ……………………………..2  runinga…………………………….3  Mabango…………………………..4  Vipeperushi /mabango/ Machapisho mengine…5  Wahudumu wa afya……………….6  Familia /marafiki/majirani…………7  Viongozo wadini /siasa/jamii………8  Walimu……………………………..9  Nyingine(Taja)…………………….10 |
| **F5** | Unapata wasiwasi gani unapofikiria kuhusu kifua kikuu?........................................................................................................................ | |
